# Effects of long-term norepinephrine treatment on normal immortalized ovarian and fallopian tube cells

**DOI:** 10.1038/s41598-021-93506-z

**Published:** 2021-07-12

**Authors:** Sweta Dash, Sean Yoder, Tania Mesa, Andrew Smith, Ling Cen, Steven Eschrich, Guillermo N. Armaiz-Pena, Alvaro N. A. Monteiro

**Affiliations:** 1 Cancer Epidemiology Program, H. Lee Moffitt Cancer Center and Research Institute, Magnolia Drive, Tampa, FL 1290233612 USA; 2 Cancer Biology Ph.D. Program, University of South Florida Tampa, Tampa, FL 33612 USA; 3 Molecular Genomics Core Facility, H. Lee Moffitt Cancer Center and Research Institute, Tampa, FL USA; 4 Data Sharing Core, H. Lee Moffitt Cancer Center and Research Institute, Tampa, FL USA; 5 Department of Basic Sciences, Pharmacology Division, School of Medicine, Ponce Health Sciences University and Divisions of Cancer Biology and Women’s Health, Ponce Research Institute, Ponce, PR USA

**Keywords:** Cancer, Cell biology

## Abstract

Sustained adrenergic stimulation by norepinephrine (NE) contributes to ovarian carcinoma metastasis and impairment of chemotherapy response. Although the effect of sustained NE stimulation in cancer progression is well established, less is known about its role in cancer initiation. To determine the extent to which stress hormones influence ovarian cancer initiation, we conducted a long-term (> 3 months; > 40 population doublings) experiment in which normal immortalized fallopian tube secretory (iFTSEC283) and ovarian surface epithelial (iOSE11) cell lines and their isogenic pairs containing a p53 mutation (iFTSEC283^p53R175H^; iOSE11^p53R175H^), were continuously exposed to NE (100 nM, 1 μM, 10 μM). Fallopian tube cells displayed a p53-independent increase in proliferation and colony-forming ability in response to NE, while ovarian surface epithelial cells displayed a p53-independent decrease in both assays. Fallopian tube cells with mutant p53 showed a mild loss of chromosomes and *TP53* status was also a defining factor in transcriptional response of fallopian tube cells to long-term NE treatment.

## Introduction

The sympathetic nervous system (SNS) is activated in response to a ‘perceived threat’ and triggers the classical acute ‘fight or flight’ reaction via rapid release of the catecholamine epinephrine and smaller amounts of another catecholamine, norepinephrine (NE). On the other hand, repetitive or chronic SNS activation upregulates NE more strongly than epinephrine^1^. Although acute activation of SNS can be beneficial to the body, chronic exposure to catecholamines has been shown to negatively impact neurochemical, immunological, and endocrinological functions^2^. Animal studies have shown that chronic stress leads to increased progression of different cancers such as prostate, breast, and ovarian cancer^3–5^.

Chronic exposure to catecholamines can promote the development of cancers through induction of DNA damage, inhibition of apoptosis, activation of oncogenes, regulation of the tumor microenvironment, and the immune response^1,2^. Molecular pathways such as β-arrestin-induced activation of MDM2 through the AKT pathway and activation of the ATR-p21 pathway have been implicated in catecholamine-induced inhibition of DNA damage repair, which could lead to chromosomal instability^6^. In ovarian carcinoma mouse models, chronic behavioral stress, which increases synthesis and release of NE, has been shown to promote tumor growth and angiogenesis in the tumor microenvironment^3^. Sustained adrenergic stimulation by NE contributes to ovarian carcinoma metastasis and impairment of chemotherapy response^7–9^.

Although the effect of sustained NE stimulation in cancer progression is well established, less is known about its role in cancer initiation. However, studies in humans have demonstrated that post-traumatic stress disorder, depression, and social isolation, conditions which increase SNS activation, are associated with a higher risk of developing ovarian cancer, suggesting a potential role in cancer initiation^10–14^. Thus, to determine the extent to which stress hormones influence ovarian cancer initiation, we conducted a long-term (> 3 months; > 40 population doublings) experiments in which normal immortalized fallopian tube secretory and ovarian surface epithelial cell lines in tissue culture were continuously exposed to NE. They were evaluated for changes in morphology, proliferation, colony-forming ability, number of chromosomes and transcriptomics. These cell lines represent a model of normal precursors cells that give rise to high grade serous ovarian carcinoma (HGSOC)^10–13^. Because *TP53* alterations are highly prevalent and happen early in the development of HGSOC^20,21^, we also evaluated exposure to continuous NE in fallopian tube and ovarian epithelial isogenic cell derivatives expressing a dominant-negative TP53 mutant (p.R175H).

## Materials and methods

### Cell lines

We used immortalized fallopian tube secretory epithelial cell lines, iFTSEC283, iFTSEC282^p53R175H^ and immortalized ovarian surface epithelial cells, iOSE11 (provided by Simon Gayther; Cedars Sinai, CA). These cells have been extensively characterized and are considered cell line models of precursor cells of HGSOC^15–18^. We also used iFTSEC283^p53R175H^ and iOSE11^p53R175H^ cell lines, which overexpress mutant p53, generated in the laboratory (see below).

NOSE-CM medium consisting of MCDB105 and Medium 199 (Sigma-Aldrich) (1:1) supplemented with 15% fetal bovine serum (Sigma-Aldrich), 0.5 mg/ml hydrocortisone (Sigma-Aldrich), 10 ng/ml epidermal growth factor (Thermo Fisher Scientific), 5 mg/ml insulin (Sigma-Aldrich), and 34 mg protein/ml bovine pituitary extract (Thermo Fisher Scientific) was used to culture iFTSEC283, iFTSEC282^p53R175H^, iFTSEC283^p53R175H^, iOSE11 and iOSE11^p53R175H^ cells. HEK293FT cells were cultured in DMEM (Thermo Fisher Scientific) supplemented with 10% fetal bovine serum (Sigma-Aldrich). Cell lines were periodically tested for mycoplasma. iFTSEC283, iFTSEC283^p53R175H^, iOSE11 and iOSE11^p53R175H^ cells were authenticated using short tandem repeat (STR) analysis.

### Lentivirus transduction for p53R175H overexpression

A V5 tagged pLenti6/V5-p53_R175H (Addgene plasmid 22,936, Junk Lab) plasmid was used to overexpress the p53 R175H dominant-negative mutant in iFTSEC283 and iOSE11 cells. The p53 R175H mutation in the plasmid was confirmed by Sanger sequencing. pLP1, pLP2, and pLP/VSVG ViraPower (Thermo Fisher) viral packaging vectors were used along with pLenti6/V5-p53_R175H to make virus particles in HEK293FT cells. Virus particles were used to transduce iFTSEC283 and iOSE11 cells, followed by blasticidin (2.5 µg/ml) selection. Single cells were then plated in 96 well plates to obtain single-cell clones. Expression of the p53 R175H mutant was confirmed by western blot using V5-tag Rabbit antibody (D3H8Q; Cell Signaling).

### Long-term treatment with norepinephrine

**Figure 1:**
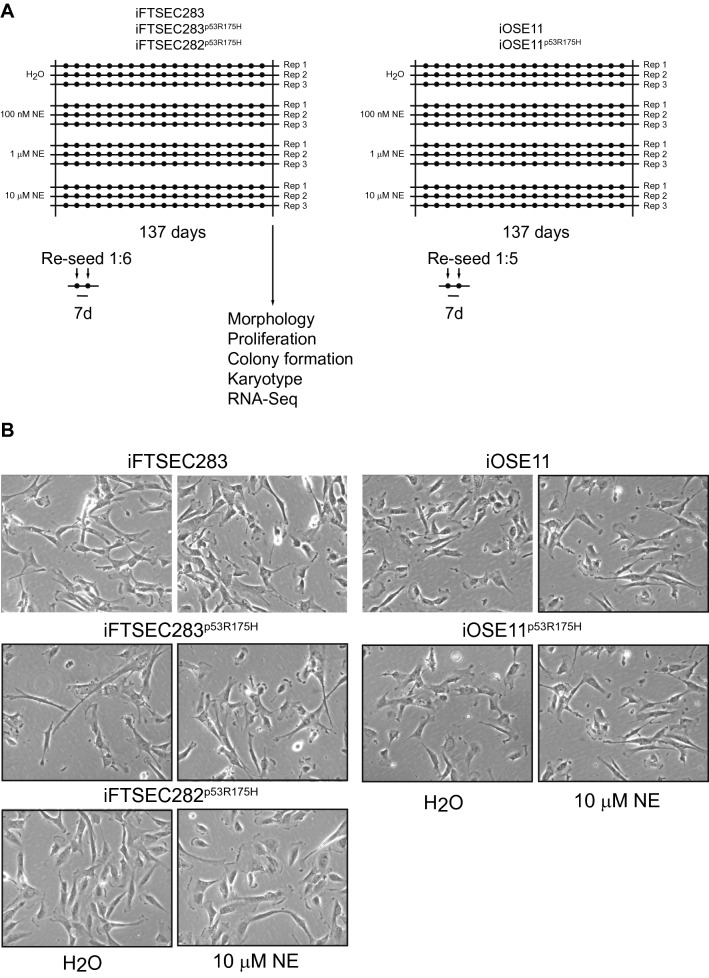
Experimental design of long-term treatment. (**A**) Diagram of the experimental design representing each independent replicate and different treatments. Black dots represent subculturing by trypsinization and re-seeding. (**B**) Phase contrast microscopy showing cell morphology under phase contrast microscopy at the end of the treatment period.

Cell lines iFTSEC283, iFTSEC283^p53R175H^, iFTSEC282^p53R175H^, iOSE11 and iOSE11^p53R175H^ were plated in 12-well plates at density of 10,000 cells per well. Each cell line had four treatment conditions: 100 nM, 1 µM, 10 µM of NE, and vehicle (H_2_O) control. Each cell line had three independent replicates with different passage numbers and plated on different days. Cells were treated for 137 consecutive days (4 ½ months). iFTSEC283, iFTSEC283^p53R175H^, iFTSEC282^p53R175H^ were re-seeded at the ratio of 1:6 every seven days and, at the end of treatment, achieved a cumulative population doubling level (PDL) of 49. iOSE11 and iOSE11p53R175H were re-seeded in the ratio of 1:5 every seven days and, at the end of treatment, achieved a cumulative PDL of 44 (Fig. 1A).

Circulating NE levels range from 0.4 to 10 nM and are increased under stress to 15 nM^19,20^. NE represents the largest fraction of ovarian catecholamines^21^ and concentrations increase markedly in pre-ovulatory follicles^22^. NE can reach local levels up to 45 ng/ml in the vesicular fraction in the ovaries of experimental models^23^. We thus assessed the effects of NE at three concentrations representing the higher end of concentrations (i.e. 100 nM, 1 µM, 10 µM) that ovarian cells might be exposed ^24,25^.

To our knowledge, there is no available information about the stability of NE in tissue culture. Studies of stability of epinephrine and NE solution in storage conditions suggest that NE is stable (defined as 90% of the drug) for 28 days at room temperature when protected from light; and approximately 90% of the initial concentration remains after seven days at 26.6 °C^26^.

We therefore chose to replace medium with fresh media containing either vehicle control or the three concentrations of NE (Sigma-Aldrich) were added to the cells (after removing old media) every two days. After this treatment, cells were referred to as long-term NE-treated cells (LTNE) or mock-treated cells.

### Proliferation assay

Three independent replicates of LTNE and mock-treated cells were seeded in 6-well plates at a density of 500 cells per well. Each replicate was seeded on a different day over a three-day period; therefore, each condition had three biological replicates. For each independent biological replicate, there were two technical replicates (total n = 6). They were then treated with the same concentration of NE they had been treated during the long-term treatment (100 nM, 1 μM, or 10 μM NE or vehicle control [H_2_O]) every alternate day until one of the wells became 90% confluent. Cells were then trypsinized and counted using Trypan blue and hemocytometer.

### Colony forming assay

Three independent replicates of LTNE and mock-treated cells were seeded in 6-well plates at a density of 200 cells per well. For each independent biological replicate, there were two technical replicates (total n = 6). Each replicate was seeded on a different day over a three-day period. They were then treated with 100 nM, 1 μM, and 10 μM NE or vehicle control (H_2_O) every alternate day for 11 days. Colonies of cells were fixed in methanol for 20 min. After removing methanol, 0.5% crystal violet solution made in 20% methanol was added to the plates and incubated for 30 min. Plates were then rinsed with dd H_2_O until the color no longer came off during rinsing and dried overnight.

### Karyotyping

LTNE (10 µM) iFTSEC283 and iFTSEC283^p53R175H^ cells and their respective mock-treated cells were split at a 1:10 ratio two days prior to karyotyping. Colchicine (0.02 μg/ml; Sigma-Aldrich) was added to the cells and incubated at 37 °C for 3 h. Cells were scraped and washed with PBS, followed by gentle addition of hypotonic solution (0.075 M KCl in H_2_O). After 15 min of incubation at 37 °C, cells were centrifuged (1200 rpm), and the pellet was re-suspended in fixative (3:1 methanol:glacial acetic acid). This step was repeated three times. Next, cells were placed onto clean slides and air-dried in a humidifying chamber to enable optimal spreading. The metaphase spreads were imaged at 900 × magnification in an inverted microscope, and chromosomes in each spread were manually counted.

### RNA isolation

LTNE (10 μM) iFTSEC283 and iFTSEC283^p53R175H^ cells and their respective mock-treated control cells were grown to reach 80% confluence on a 100 mm plate. Cells were harvested and processed for total RNA extraction using the RNeasy Plus Mini Kit (Qiagen, Hilde, Germany) following the manufacturer’s protocol, which includes removal of genomic DNA by gDNA Eliminator columns. The samples' ratio of absorbance at both 260/280 and 260/230 was ≥ 2 as measured by Nanodrop. The isolated RNA samples were used for sequencing.

### Library preparation and sequencing

Total RNA (100 ng) was isolated from three independent replicates for each condition: LTNE (10 μM) iFTSEC283 and iFTSEC283^p53R175H^ cells and their respective mock-treated control cells. Twelve libraries were prepared using the Nugen Universal RNA Seq Kit (NuGEN Technologies, San Carlos, CA). Illumina NextSeq500 instrument was used for sequencing with 75 bp paired-end reads. Approximately 28 million pairs of reads for each sample were generated on average, and the average alignment rate was ≥ 94.2% (Supp. Table 1).

### RNA-sequencing analysis

Sequencing reads were aligned against human reference genome hs37d5 using TopHat2. HTSeq with Gencode v19 was used to determine gene-level quantification by summation of raw counts of reads aligned to the region associated with each gene. DESeq2 was used for library size normalization and differential expression analysis. Sequencing depth, gene length, and RNA composition were considered for normalization and differential expression analysis. iFTSEC283 and iFTSEC283^p53R175H^ cells were normalized separately, and differential expression analysis was performed on NE-treated vs. mock-treated cells for the two cell lines. Significantly differentially expressed genes were determined using p_adj_ (p-value adjusted for multiple testing with the Benjamini–Hochberg correction) of less than 0.1.

Data for the RNA-Seq experiments described here are available through NCBI Gene Expression Omnibus (GSE168097).

### Regulatory motif enrichment analysis

oPOSSUM single-site analysis was applied to identify transcription factor binding sites enriched in our input gene set: differentially expressed genes (FDR < 0.1) obtained from RNA sequencing analysis in iFTSEC283 and iFTSEC283^p53R175H^ cell lines. We used ‘Single Site Analysis’ to identify transcription factor binding sites (TFBS) enriched in 5 kb sequence upstream and downstream of the transcription start site. We ranked the enriched TFBS using two complementary statistical models: Fisher test, a one-tailed probability test comparing the proportion of the target gene set containing a TFBS to background (http://opossum.cisreg.ca/oPOSSUM3/help.html#fisher), and Z-score, a two-tailed analysis that uses the normal approximation to the binomial distribution to compare the rate of occurrence of a TFBS in the target gene set to the expected rate estimated from the background set (http://opossum.cisreg.ca/oPOSSUM3/help.html#zscore). The following options were used for oPOSSUM analysis: 85% matrix match threshold, sequences of − 5,000 to + 5,000 bp from the transcription start site, 0.40 conservation cutoff, and all genes in the oPOSSUM database.

### Gene ontology and pathway analysis

Gene Ontology (GO) enrichment analysis was performed using PANTHER (Version 15.0 released 2020-02-14) Statistical Overrepresentation Test. Binomial test type and False Discovery Rate correction (FDR < 0.05) were applied.

### Western blotting

LTNE and mock-treated cells were cultured up to 80% confluence in 100 mm plates. Cells were harvested by scraping followed by extraction of the cytoplasmic fraction by incubation for 2 min on ice in lysis buffer A [20 mM Tris pH 7.4, 10% glycerol, 10 mM KCL, 0.2% NP-40, 1 mM EDTA, 0.6 mM β-mercaptoethanol] supplemented with 1 × protease inhibitor cocktail (Roche, Basel, Switzerland) and 1 mM PMSF. Following centrifugation (12,000 rpm) at 4 °C, the supernatant containing the cytoplasmic fraction was collected and the pellets were re-suspended in nuclear extract buffer B [20 mM Tris (pH 7.4), 20% glycerol, 10 mM KCL, 0.4 M NaCl, 1 mM EDTA, 0.6 mM β-mercaptoethanol, 1 mM PMSF and 1 × protease inhibitor cocktail]. Resuspended cells were incubated for 30 min on ice. Bradford Assay (Bio-Rad Laboratories, Hercules, California) was used to determine protein concentration. Whole cell﻿ lysates containing 50 µg of both cytoplasmic and nuclear fractions were resolved in 10% polyacrylamide gels and transferred to methanol-activated PVDF using the TransBlot Turbo system (Bio-Rad Laboratories, Hercules, California). Antibodies: V5-tag (Cell Signaling; dilution 1:1000).

### qPCR

cDNA was synthesized from isolated RNA using Qiagen QuantiTect Reverse Transcription Kit with genomic DNA removal. PowerUp™ SYBR™ Green Master Mix (Thermo Fisher Scientific) was used for performing gene expression analysis of the following genes: *ADRB2*, *CDKNA1, PUMA, APOBEC3C, TP53I3, NPTX1, SCD, PTGES, MCAM, PLAU, PLAC8, DSP, ABI3BP, POSTN, BGN, MAPK13, LRRC17* and *RCAN2*, with β-actin as an internal control. Analysis was done on two independent replicates and each had three technical replicates (total n = 6). Expression for each gene of interest was calculated as a relative expression ratio normalized to *ACTB* (β-actin) expression levels. The Δ-Δct method was used for calculating the relative expression of genes compared to mock-treated cells.

## Results

### Generation of isogenic iFTSEC283 cells overexpressing mutant p53

To explore the effects of chronic exposure to NE on precursor cells of HGSOC, we chose well-characterized immortalized fallopian tube secretory epithelial cells, iFTSEC283, and ovarian surface epithelial cells iOSE11. To model early changes in ovarian cancer development, we generated iFTSEC283 and iOSE11 cells overexpressing a dominant-negative *TP53* mutant (p.R175H) because p53 alterations are the earliest and most prevalent in HGSOC. Cells were transduced with a V5-tagged p53R175H cDNA, and single-cell clones were selected. Expression of the mutant p53 was confirmed by western blotting (Supp. Figure 1A).

### Long-term treatment with norepinephrine

Five cell lines (iFTSEC283, iFTSEC283^p53R175H^, iFTSEC282^p53R175H^, iOSE11 and iOSE11^p53R175H^) were continuously cultured with either vehicle control (H_2_O) or three concentrations of NE (100 nM, 1 µM or 10 µM) for 137 days (Fig. 1A). After 137 days, no change in cell morphology was observed in any cell lines, even at the highest concentration of NE (Fig. 1B). There was no significant difference in the levels of the Beta-2 Adrenergic Receptor (*ADRB2)* (Supp. Figure 1B).

### Long-term NE treatment leads to increased proliferation and colony formation in fallopian tube cell lines

**Figure 2:**
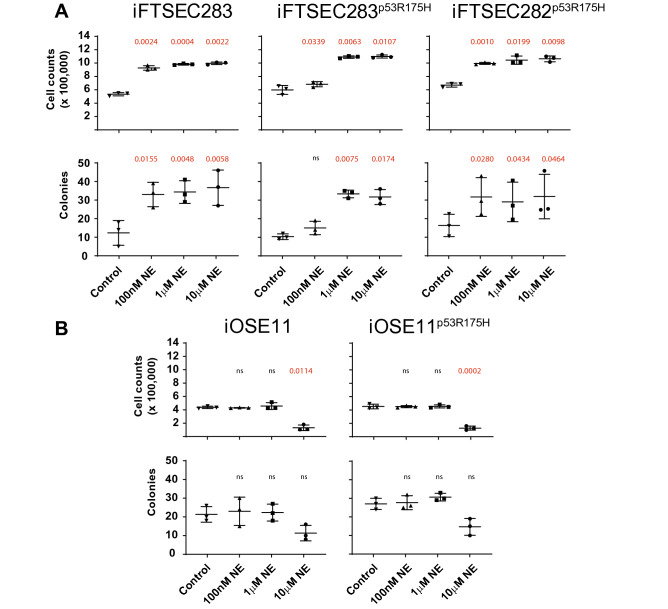
Effect of long-term exposure to NE on cell survival. (**A**) Short-term proliferation and (**B**) colony forming assays capacity of cells treated with NE (100 nM, 1 µM, 10 µM) or mock treated for 4 months. Short-term proliferation and colony forming assays were conducted with the same concentrations (or mock treatment) as the long-term treatment. Statistical significance (p value) in a paired t-test in relation to control is indicated. ns, not significant.

All three fallopian tube cell lines (iFTSEC283, iFTSEC283^p53R175H^, and iFTSEC282^p53R175H^) showed an increase in proliferation after four months of treatment with 1 µM or 10 µM NE compared to vehicle control, but only two cells lines (iFTSEC283 and iFTSEC282^p53R175H^) displayed increased proliferation at 100 nM NE (Fig. 2A). In contrast, the ovarian surface epithelial cells, iOSE11 and iOSE11^p53R175H^, showed decreased proliferation when treated with 10 µM NE for four months compared to vehicle control, but no difference in the proliferation capacity was observed between control-treated cells vs. cells exposed to 1 µM or 100 nM NE (Fig. 2B).

Consistent with the proliferation patterns, the three fallopian tube cell-lines showed increase capacity for colony formation after chronic exposure to NE at 1 µM or 10 µM NE compared to vehicle control, but only two cells lines (iFTSEC283 and iFTSEC282^p53R175H^) displayed increased colony forming ability at 100 nM NE (Fig. 2A). In contrast, the two ovarian surface epithelial cell lines demonstrated no significant difference in colony-forming capacity in treated vs. vehicle control (Fig. 2B) (Supp. Figure 2).

### Decreased fraction of diploid metaphases in p53R175H-overexpressing fallopian tube cells

Because fallopian tube cell lines displayed increased proliferation rates (Fig. 2A), we hypothesized that long-term treatment could cause genomic instability. We performed karyotyping using solid Giemsa stain to assess chromosomal structural and number abnormalities in long-term treated (10 µM NE) and mock-treated fallopian tube iFTSEC283 and iFTSEC283^p53R175H^ cells.

**Figure 3:**
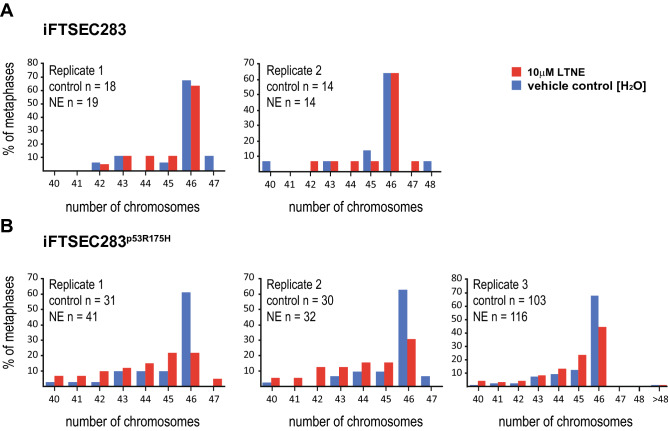
Effect of long-term exposure to NE on chromosome number. (**A**) Percentage of metaphase containing the indicated number of chromosomes in 10 µM NE (red bars) and mock-treated (blue bars) iFTSEC283 cells. (**B**) Percentage of metaphase containing the indicated number of chromosomes in 10 µM NE (red bars) and mock-treated (blue bars) iFTSEC283^p53R175H^ cells. Absolute number of metaphases assessed in each condition is also shown.

Mock-treated iFTSEC283 and iFTSEC283^p53R175H^ cells displayed ~ 65% [range 61–67%] of diploid metaphases (n = 46) (Fig. 3). Long-term treatment (10 µM NE) did not affect the fraction of diploid metaphases (63–64%) in iFTSEC283. In contrast, long-term treatment iFTSEC283^p53R175H^ cells displayed a decrease in the fraction of diploid metaphases (~ 33%; range 22–44%) with a corresponding increase in the fraction of sub-diploid metaphases (Fig. 3). No gross abnormalities (e.g., tri-radials, quadri-radials, chromosome fusions) were observed.

### Transcriptomic profile generated by RNA-Seq

Next, we performed transcriptomic analysis following RNA sequencing on iFTSEC283 and iFTSEC283^p53R175H^ cell lines long-term treated with 10 µM NE or mock-treated to identify genes that are differentially expressed by long-term NE treatment in p53^+^ and in p53^-^ backgrounds.

**Figure 4:**
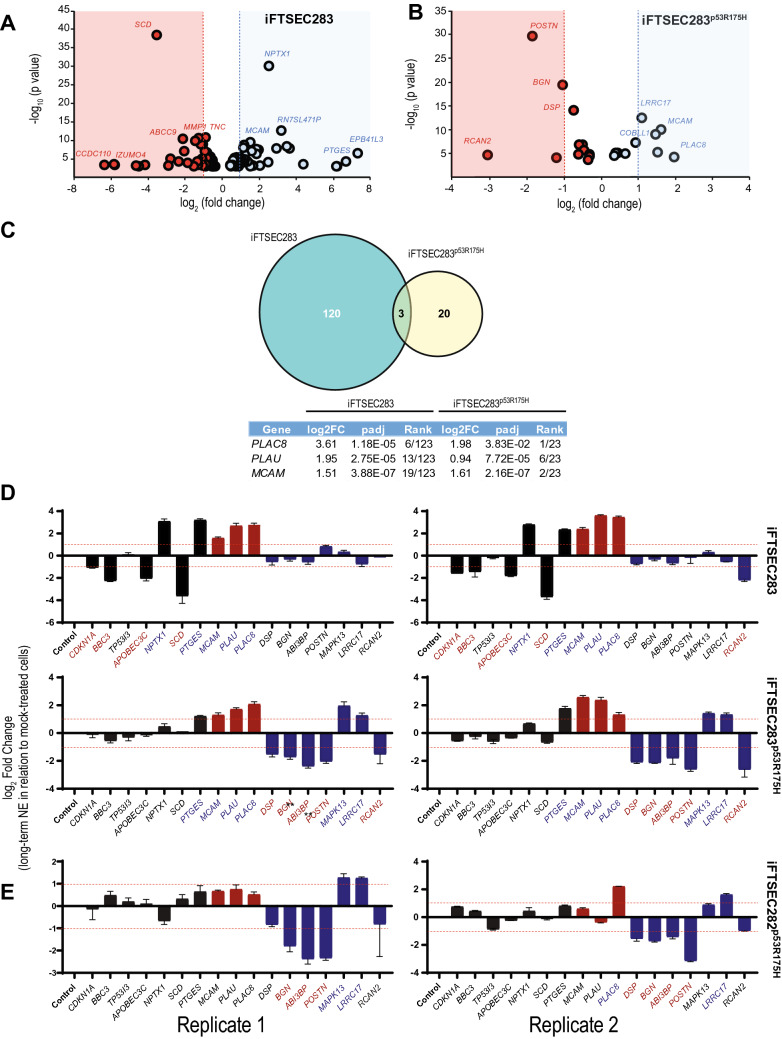
Transcriptomic profile and Regulatory Motif Enrichment Analysis. Volcano plots of genes differentially expressed in (**A**) iFTSEC283 and (**B**) iFTSEC283^p53R175H^ cells (mock versus 10 μM NE treatment). (**C**) Venn diagram showing differentially expressed genes in response to chronic norepinephrine (NE) treatment in iFTSEC283 and iFTSEC283^p53R175H^ cells. (**D**) qPCR validation of RNA-Seq data in iFTSEC283 and iFTSEC283^p53R175H^ cells. Genes considered to be differentially (− 1 > Log2FoldChange > 1; *p* < 0.05) up and down regulated are denoted by blue and red font, respectively. Bars are colored according to (**a**) genes differentially expressed (FDR < 0.1) in iFTSEC283 cells (black bars); (**b**) the three genes differentially regulated (FDR < 0.1) irrespective of p53 status (red bars); and (**c**) and seven genes differentially expressed (FDR < 0.1) in iFTSEC283^p53R175H^ cells (blue bars).(** E**) qPCR validation of RNA-Seq data in iFTSEC282^p53R175H^ cells. Genes considered to be differentially (− 1 > Log2FoldChange > 1; *p* < 0.05) up and down regulated are denoted by blue and red font, respectively.

In iFTSEC283 cells, 123 genes were differentially expressed in cells treated for long term with 10 µM NE when compared to mock-treated cells (FDR < 0.1) (Fig. 4A) (Supp. Table 2). Four known p53 target genes were differentially regulated in iFTSEC283 cells compared to mock-treated cells, but not in their p53-mutant counterpart, after long-term 10 µM NE treatment (https://p53.iarc.fr/TargetGenes.aspx). *CDKN1A* (p21), *BBC3* (PUMA), and *APOBEC3C* were downregulated, and *TP53I3* was upregulated in response toLTNE treatment in iFTSEC283 cells compared to mock-treated cells (Supp. Tables 3 and 4).

In iFTSEC283^p53R175H^ cells, 23 genes were differentially expressed in cells treated long-term with 10 µM NE versus mock-treated cells. Five upregulated (*PLAC8*, *MCAM*, *MAPK13*, *COBLL1,* and *LRRC17*) and 4 downregulated (*RCAN2*, *POSTN*, *ABI3BP,* and *BGN*) genes had ≥ twofold change in expression (Supp. Table 5) (Fig. 4B). Consistent with the ectopic overexpression of mutant p53, *TP53* levels were 18X higher in iFTSEC283^p53R175H^ cells in both treatment conditions than in iFTSEC283 cells of the same condition (Supp. Tables 3 and 4and the p53 target genes (found to be differentially regulated in iFTSEC283 cells compared to mock-treated cells) were not differentially expressed, after long-term 10 µM NE treatment.

Three genes were differentially expressed in cells treated with 10 µM NE independent of p53 background (i.e. they were differentially expressed in long-term NE treated iFTSEC283 and iFTSEC283^p53R145H^ when compared to their mock-treated controls): *PLAC8*, *PLAU,* and *MCAM*. All three genes were upregulated and were among the most highly differentially expressed genes in relation to mock-treated cells (Fig. 4C). The low number of overlapping genes between iFTSEC283 and iFTSEC283^p53R145H^ suggests that p53 status is a critical determinant of the cellular response to NE.

### Gene ontology and pathway analysis

Panther database was used to perform Gene Ontology analysis on differentially expressed genes identified by RNA-seq in iFTSEC283 and iFTSEC283^p53R175H^ cells when comparing mock- to long-term 10 µM NE treatment.

In iFTSEC283 cells, GO Biological Process analysis of the upregulated genes in NE treated cells showed enrichment of 36 biological processes (Supp. Table 6). The most significantly enriched processes (Fold enrichment ≥ 3) were regulation of apoptotic process (GO:0,042,981) and regulation of programmed cell death (GO:0,043,067) (Supp. Table 6). GO Cellular Component analysis revealed ‘nucleosome (GO:0,000,786)’ and ‘DNA packaging complex (GO:0,044,815)’ to be significantly overrepresented (Supp. Table 7), and the only Panther Protein Class to be significantly enriched (Supp. Table 8) was the histone (PC00118) class with a 28.61-fold enrichment. Interestingly, five histone transcripts were upregulated in response to NE in iFTSEC283 cells: *HIST1H3J*, *HIST1H2BE*, *HIST1H2BB*, *HIST1H2AL*, *HIST1H2AH* (Supp. Table 2). In addition, the most significantly overrepresented Reactome Pathway was HDACs deacetylate histones (R-HSA-3214815) (Supp. Table 9). These results suggest that long-term NE treatment modulates the epigenetic state of iFTSEC283 cells. GO biological process and Reactome Pathway analysis of the downregulated genes in iFTSEC283 cells showed overrepresentation of processes and pathways involved in lipid metabolism and steroid biosynthesis (Supp. Tables 10 and 11).

In iFTSEC283^p53R175H^ cells, analysis of the 23 differentially expressed genes according to GO biological process, molecular function, and cellular component revealed processes involved in extracellular structure and matrix organization to be significantly overrepresented (Supp. Table 12–14). Many of the genes involved in these processes were downregulated in response to long-term treatment with 10 μM NE, including intercellular tight junction component desmoplakin (*DSP*), collagen interacting proteoglycan biglycan (*BGN*), extracellular matrix protein ABI3 binding protein (*ABI3BP*), and integrin binding protein periostin (*POSTN*) (Supp. Table 5) (Fig. 4B). Analysis of only the upregulated genes did not show any significantly overrepresented process or pathway.

### Regulatory motif enrichment analysis

To identify gene regulatory mechanisms induced by long-term exposure to NE, we performed transcription factor enrichment analysis on the 123 and 23 genes differentially expressed in iFTSEC283 and iFTSEC283^p53R175H^ cells, respectively, compared to mock-treated cells using the oPOSSUM database^27^.

In iFTSEC283 cells, we identified four enriched transcription factors with a Z-score higher than two standard deviations above the mean: MZF1_1-4, Pax4, Myc, and Klf4 (Supp. Table 15) (Supp. Figure 3A). When ranked by Fisher scores, 17 transcription factors had a score higher than one standard deviation above the mean, with transcription factors Myc and Klf4 also being identified (Supp. Figure 3B).

In iFTSEC283^p53R175H^ cells, we identified transcription factor FOXA1 among the top three enriched transcription factors when ranked by both z-score and Fisher Score (Supp. Figure 3A) (Supp. Table 16). In addition, when the TFBS enrichment was ranked based on Fisher-score, Myc::Max heterodimer was among the 19 transcription factors that had a Fisher score higher than one standard deviation above the mean (Supp. Figure 3B).

The only commonality between the two cell lines was the enrichment of genes with Myc binding sites in their promoters in iFTSEC283 cells and with Myc::Max in iFTSEC283^p53R175H^. A total of 68 differentially expressed genes in iFTSEC283 cells contain predicted Myc binding sites, including *CDKN1A*, *HIST1H2BB*, *MEG3,* and *PLAU* (Supplementary Table 17). In iFTSEC283^p53R175H^, eight differentially expressed genes contain predicted Myc::Max binding sites (*PLAU*, *CITED2*, *CYR61*, *EGR1*, *SCARA3*, *DSP*, *ABI3BP,* and *LRRC17*). Reflecting the low overlap between the two isogenic cell lines in differentially expressed genes upon NE treatment, enriched transcription factors were also largely distinct and support the notion that p53 status is an important determinant of the cellular response to NE.

### Validation of differentially expressed genes

To validate the pattern of differentially expressed genes upon long-term NE treatment with an alternative method, we tested gene expression in mock-treated and long-term [10 μM] NE treated iFTSEC283 and iFTSEC283^p53R175H^ cells by qPCR for several genes. First, we assessed the seven genes differentially expressed (FDR < 0.1) in iFTSEC283 cells, including *PTGES* and *NPTX1* (among the 10 most upregulated genes), *SCD* (a top downregulated gene), and four p53 target genes (*CDKN1A*, *BBC3*, *APOBEC3C* and *TP53I3)*. Second, we assessed the three genes (*PLAU, MCAM, PLAC8)* differentially regulated (FDR < 0.1) irrespective of p53 status (Fig. 4C), and third, seven of the top 5 most up- or downregulated genes (FDR < 0.1, *MAPK13, LRRC17, DSP*, *BGN*, *ABI3BP*, *POSTN, RCAN2*) in iFTSEC283^p53R175H^ cells. We considered a gene to be differentially regulated when levels of expression in mock-treated cells were significantly (p < 0.05) different from long-term NE treated cells and the Log2 fold change was < -1 or > 1.

Overall, 13/17 (76%) of the genes chosen for validation were consistent across qPCR and RNA-Seq results. Consistent with the RNA-seq results, *PTGES* and *NPTX1* were upregulated and *CDKN1A* (coding for p21), *BBC3* (coding for p53-upregulated modulator of apoptosis (PUMA)), *APOBEC3C* and *SCD* were downregulated in long-term [10 μM] NE treated iFTSEC283 cells compared with control in independent replicates using qPCR (Fig. 4D, black bars); while in NE treated iFTSEC283^p53R175H^, with the exception of *PTGES*, the expression of these genes remained unaltered compared with mock-treated controls (Fig. 4D, black bars). Expression levels of p53 target genes (*CDKN1A*, *BBC3* and *APOBEC3C*) were significantly lower in mock treated iFTSEC283^p53R175H^ compared to mock treated iFTSEC283 cells and treatment with NE did not further alter their levels (Supp. Figure 4). Results for *TP53I3* were inconsistent with the RNA-Seq data, with qPCR expression levels being unaltered in IFTSEC283 cells (Fig. 4D, black bars). The three genes differentially regulated irrespective of p53 status, *MCAM, PLAC8* and *PLAU* were shown to be significantly upregulated in iFTSEC283 and iFTSEC283^p53R175H^ in both replicates (Fig. 4D, red bars) consistent with RNA-Seq data. Differential gene expression for *DSP*, *BGN*, *ABI3BP, POSTN, MAPK13,* and *LRRC17* was also consistent with the RNA-seq data (Fig. 4D, blue bars). Results for *RCAN2* which was downregulated in iFTSEC283^p53R175H^ cells and in one replicate of iFTSEC283 cells were inconsistent with the RNA-Seq results (Fig. 4D, blue bars). Additionally, *PTGES* was found to be upregulated in iFTSEC283^p53R175H^ cells in qPCR, but not in RNA-Seq.

Finally, to explore how robust these changes were across different cell lines, we performed qPCR gene expression analysis on iFTSEC282^p53R175H^ cells. Six out of the seven genes differentially expressed in iFTSEC283 cells only showed no significant change in expression in either p53R175H cell lines (Fig. 4D–E; compare black bars). However, the three genes differentially regulated irrespective of p53 status were not regulated in iFTSEC282^p53R175H^ (Fig. 4D–E; compare red bars). Finally, the seven genes differentially expressed in iFTSEC283^p53R175H^ cells behaved similarly in iFTSEC282^p53R175H^ (Fig. 4D–E; compare blue bars). Although limited to a few select genes, the data suggest a strong similarity (13/17 genes tested) between the p53 mutant expressing cells but not with p53 wild type cells.

## Discussion

Psychosocial stress has been associated with cancer progression in several settings^3–5^. In this report, we evaluated the long-term in vitro effects of norepinephrine (NE) treatment using a model of isogenic cells postulated to be the precursors of ovarian cancer—the fallopian tube epithelial cells (iFTSEC283) and ovarian surface epithelial cells (iOSE11)^15–18^, which share tissue-specific signatures and transcription regulatory architecture^28^, and isogenic cell lines with oncogenic dominant-negative mutant p53 p.R175H to assess the extent to which p53 status influences transcriptional responses after long-term exposure to NE. We assessed the effects of NE at three concentrations representing the higher end of concentrations that ovarian cells might be exposed^24,25^.

Overall, we observed no morphological changes in iFTSEC283, iFTSEC283^p53R175H^, iFTSEC282^p53R175H^, iOSE11 and iOSE11^p53R175H^ cells, although long-term treatment with 1 µM and 10 µM NE increased proliferation and colony-forming capacity of the fallopian tube epithelial cells with and without p53 mutation. In contrast, ovarian surface epithelial cells showed reduced proliferation and colony-forming capability, even in the p53 mutant background. This suggests that fallopian tube cells may be more susceptible to oncogenic effects of NE than ovarian surface epithelial cells. This is consistent with epidemiologic studies suggesting that Posttraumatic Stress Disorder (PTSD) was more strongly associated with increased risk of high-grade serous ovarian cancer, than ovarian cancer overall^10^. Notably, PTSD patients display higher concentrations of epinephrine and norepinephrine than normal controls^29–31^.

Interestingly, a decrease in the percentage of metaphase spreads containing 46 chromosomes was observed after chronic 10 µM NE treatment only in cells expressing mutant p53. These results suggest that NE and oncogenic p53 alterations can act in combination to promote chromosomal number changes. Several studies have shown the association of chromosomal instability (CIN) with epithelial ovarian cancer. HGSOC is characterized by highly abnormal karyotypes along with other features of genomic instability including numerical and structural variants^32–35^. CIN has also be demonstrated in ovarian cancer cell lines and in ascites derived from patients with HGSOC and several underlying mechanisms have been suggested, including DNA replication stress and elevated microtubule dynamics^33,36^.

Transcriptomic profiling of 10 µM LTNE in iFTSEC283 and iFTSEC283^p53R175H^ cells revealed very low overlap in gene expression compared to mock-treated controls between the two cell lines, with only three genes differentially expressed (*PLAC8, PLAU, MCAM),* suggesting that p53 status is a critical determinant of the response to NE. Interestingly, sympathetic neurons release PLAU, a urokinase plasminogen activator, which appears to be involved in proliferation and migration of follicular cells and extra cellular matrix degradation and angiogenesis in corpus luteum formation^37,38^. Further, *PLAU* overexpression is commonly observed in ovarian tumors and is associated with higher stage and grade as well as poor outcomes, demonstrating potential biologic mechanisms linking NE to ovarian carcinogenesis^39^.

In wild type p53 iFTSEC283 cells, known p53 target genes, *CDKN1A* (coding for p21), *BBC3* (coding for PUMA), and *APOBEC3C* were downregulated in response to NE^40^. Both *CDKN1A* and *BBC3* genes are induced by p53, usually in response to DNA damage, to cause cell cycle arrest and apoptosis, respectively. Deregulation of these genes leads to loss of cell cycle arrest and apoptosis inhibition, even after DNA damage^41,42^. *APOBEC3C* belongs to the DNA cytosine deaminases family of proteins required for mRNA editing (C to U conversion). In addition to p53 target genes, five histone transcripts were upregulated by long-term exposure to NE in iFTSEC283 cells. Gene Ontology Reactome Pathway analysis revealed an overrepresentation of HDACs, DNA methylation, HATs, and Chromatin modifying pathways suggesting that NE may play a role in epigenetic regulation of iFTSEC283 cells. One of the most strongly upregulated genes, prostaglandin E synthase (*PTGES*) (log2FoldChange = 6.67), was shown to contribute to ovarian carcinoma by increasing metastasis through prostaglandin E2 (PGE2) synthesis via ADRB2-Nf-kB axis^7^.

In iFTSEC283^p53R175H^ cells, transcriptomic profile revealed genes involved in an extracellular matrix organization to be differentially regulated. These genes include downregulation of intercellular tight junction component desmoplakin (*DSP*), collagen interacting proteoglycan biglycan (*BGN*), extracellular matrix protein ABI3 binding protein (*ABI3BP*), and integrin binding protein periostin (*POSTN*); and upregulation of extracellular matrix-degrading urokinase *PLAU* and cell adhesion molecule *MCAM*. Additionally, in response to chronic NE treatment, iFTSEC283^p53R175H^ cells also upregulated a stress-activated p38 isoform, *MAPK13*, which has been shown to be positively associated with tumor initiation ^43^.

A comparison with the transcriptomic data set of iFTSEC283 cells induced by short-term (1 h) NE treatment revealed only 11 genes (*ATOH8, CD55, EDN1, FAM167A, ID1, ID2, ID3, IRF2BP2, NPTX1, PCDH9, SIX4*) regulated in the same direction in both datasets, representing 4.7% (11/234) and 8.9% (11/123) of the differentially expressed genes, in short and long-term respectively^44^. These data suggest that the transcriptional response to acute NE treatment is distinct from long-term NE treatment.

HGOSC arises from an early noninvasive tumor lesion preferentially formed in the fallopian tube cells, and these precancerous lesions have an elevated proliferative capacity, loss of polarity, and increased p53 alterations^45–47^. Our study exploits a well-characterized model of precursor cells for HGSOC to assess the role of long-term NE exposure. The in vitro environment and the small number of cell lines studied are limitations of our study. Different from ovarian cancer cell lines for which there are over 100 lines available, there are only a few well characterized immortalized cells from human ovarian surface epithelium or fallopian tube secretory cells^15,18,28,46,47^. We also recognize that some effects may be limited to high concentrations of NE. Despite these limitations, which may limit the generalizability of our conclusions, the study provides a model to study the effects of behavioral stress on ovarian cancer initiation and development in isogenic cell lines.

Our results suggest that fallopian tube cells are more likely to respond to long-term NE treatment with increased proliferation than ovarian surface epithelial cells. In fallopian tube cells, *TP53* mutation alone or long-term NE alone were not enough to cause chromosomal changes, but their combination led to a change in the number of diploid metaphases. *TP53* status was also a strong determinant of the transcriptional response to NE, suggesting that fallopian tube cells in precursor lesions that already contain p53 mutations respond differently than *TP53* wild type cells. Additional experiments will be needed to clarify the biological relevance of the NE-induced transcriptional program, particularly the role of extracellular matrix proteins and enzymes that modify the tissue environment.

## Supplementary Information

Supplementary Information 1.

Supplementary Information 2.

## Data Availability

Data for the RNA-Seq experiments described here are available through NCBI Gene Expression Omnibus (GSE168097).
