# Supplementary material for: Effects of long-term norepinephrine treatment on normal immortalized ovarian and fallopian tube cells
Source: Sci Rep. 2021 Jul 12;11:14334. doi: 10.1038/s41598-021-93506-z (PMC8275603; doi:10.1038/s41598-021-93506-z)
Supplement: Supplementary file 1 — Supplementary Information 1. [file 41598_2021_93506_MOESM1_ESM.pdf]

Supp. Figure 1

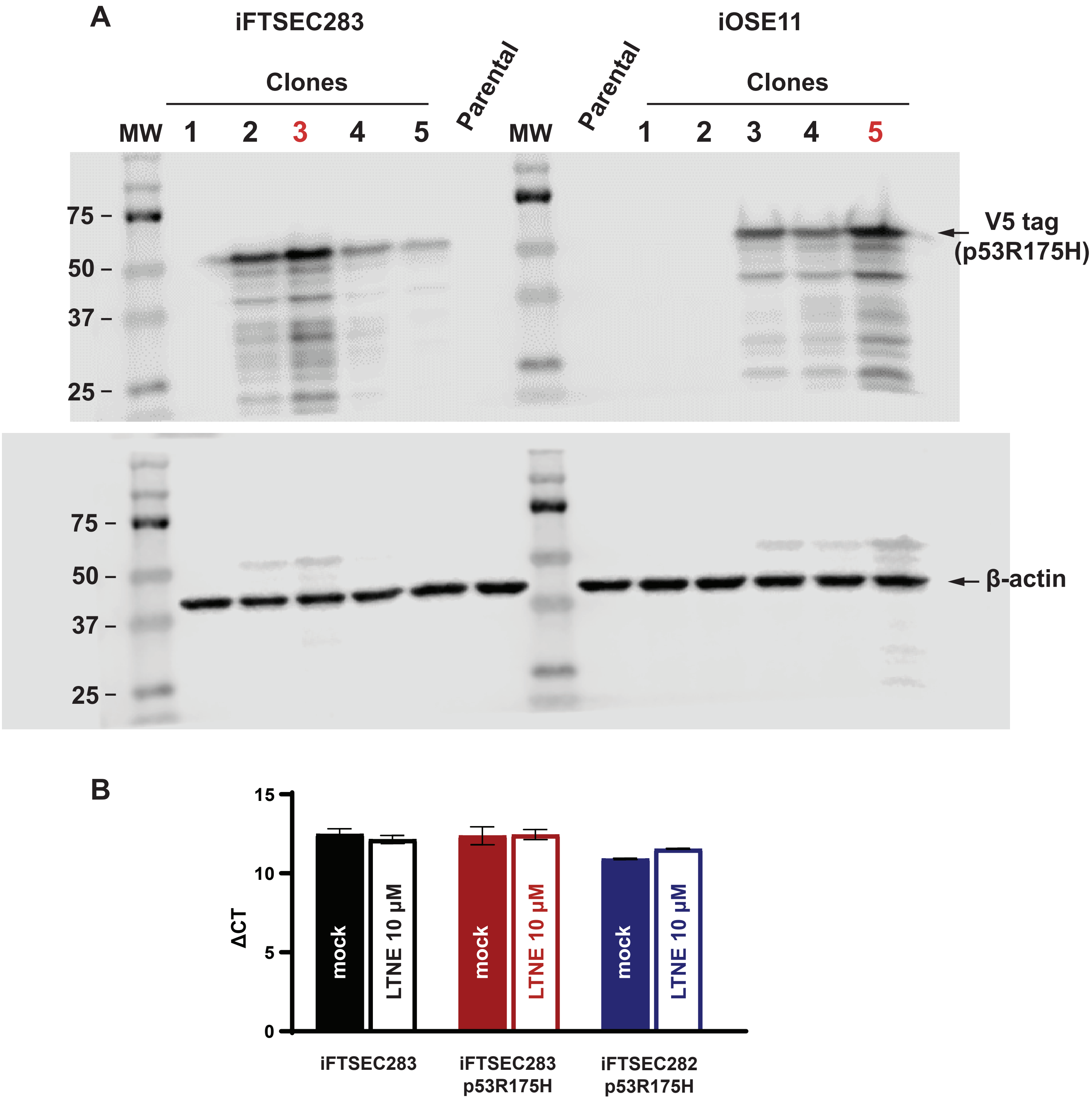

Supplementary Figure 1. A. Selection of iFTSEC283 and iOSE11 clones stably expressing V5-tagged p53 (p.R175H). Clones used for subsequent experiments are denoted by red font. **B.** Expression levels for Adrenergic beta-2 receptor gene (*ADRB2*) in mock and long-term norepinephrine exposure treated cells.

Supp. Figure 2

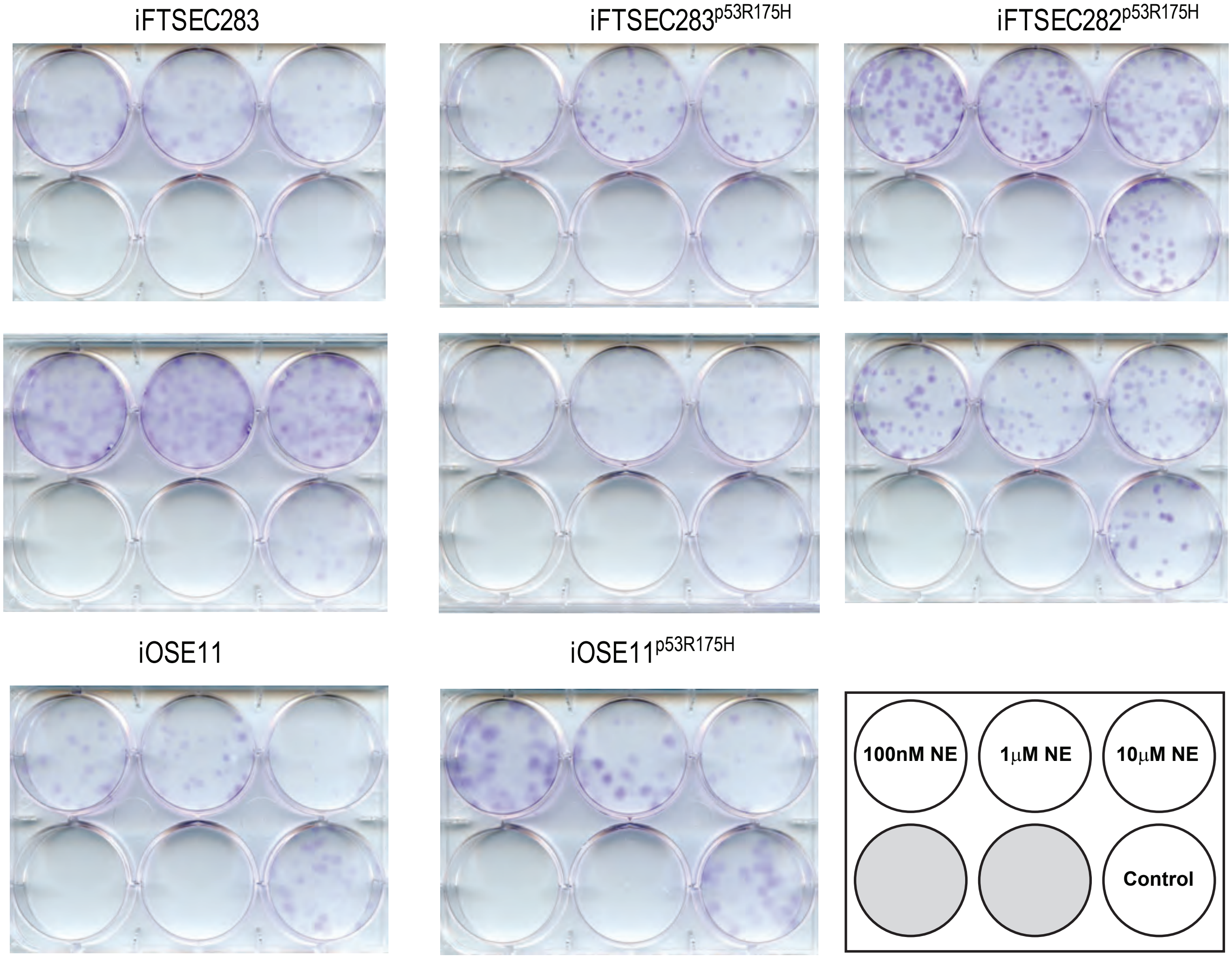

Supplementary Figure 2. Colony forming assays. LTNE and mock-treated cells were seeded in 6-well plates at a density of 200 cells per well then treated with 100 nM, 1 μM, and 10 μM NE or vehicle control (H_2O) every alternate day for 11 days. Each replicate was seeded on a different day over a three-day period. Cells were fixed in methanol and stained with 0.5% crystal violet solution made in 20% methanol.

Supp. Figure 3

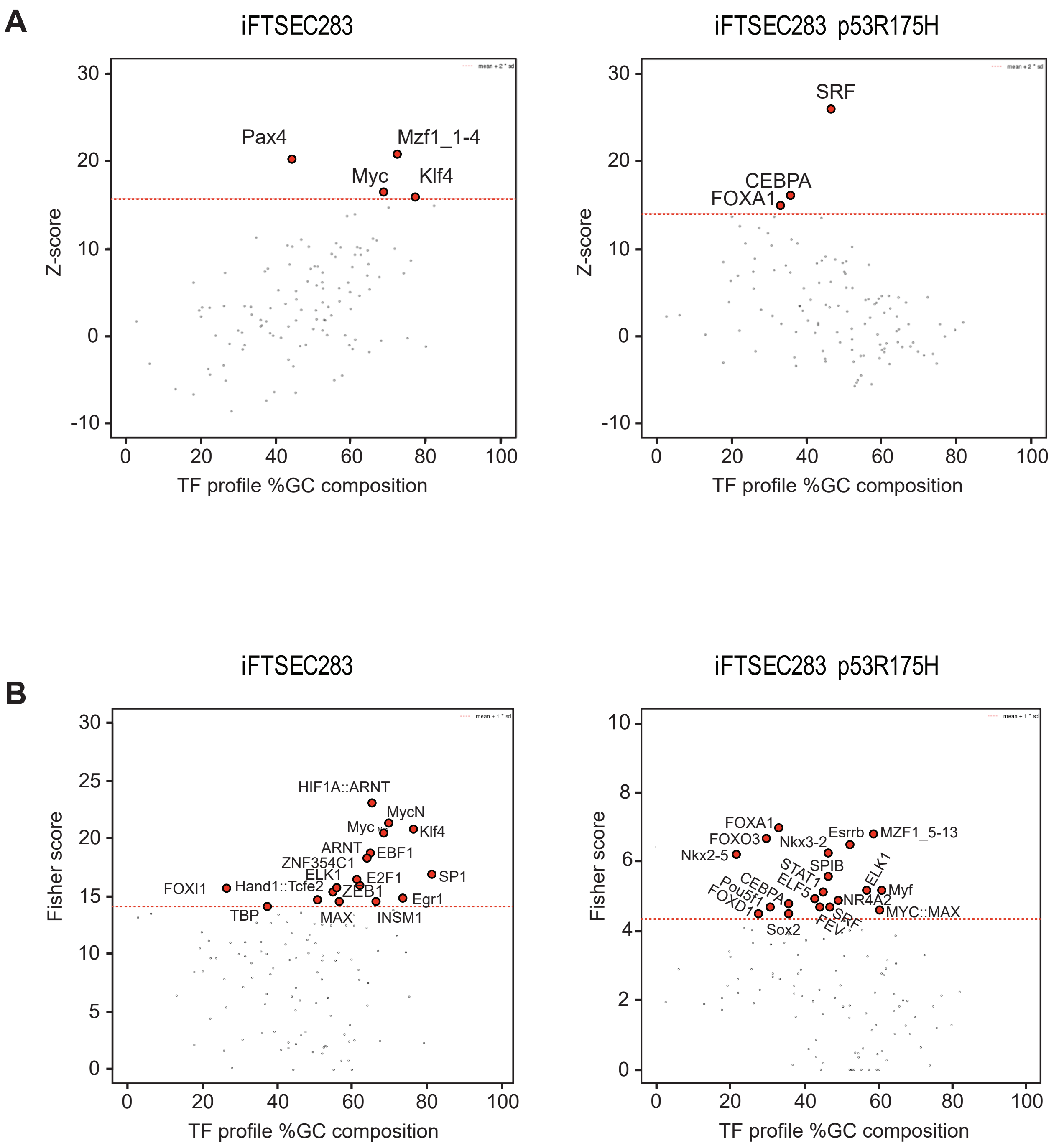

Supplementary Figure 3. Regulatory Motif Enrichment Analysis. Significantly enriched transcription factors identified using oPOSSUM by (**A**) Z-score and (**B**) Fisher score.

Supp. Figure 4

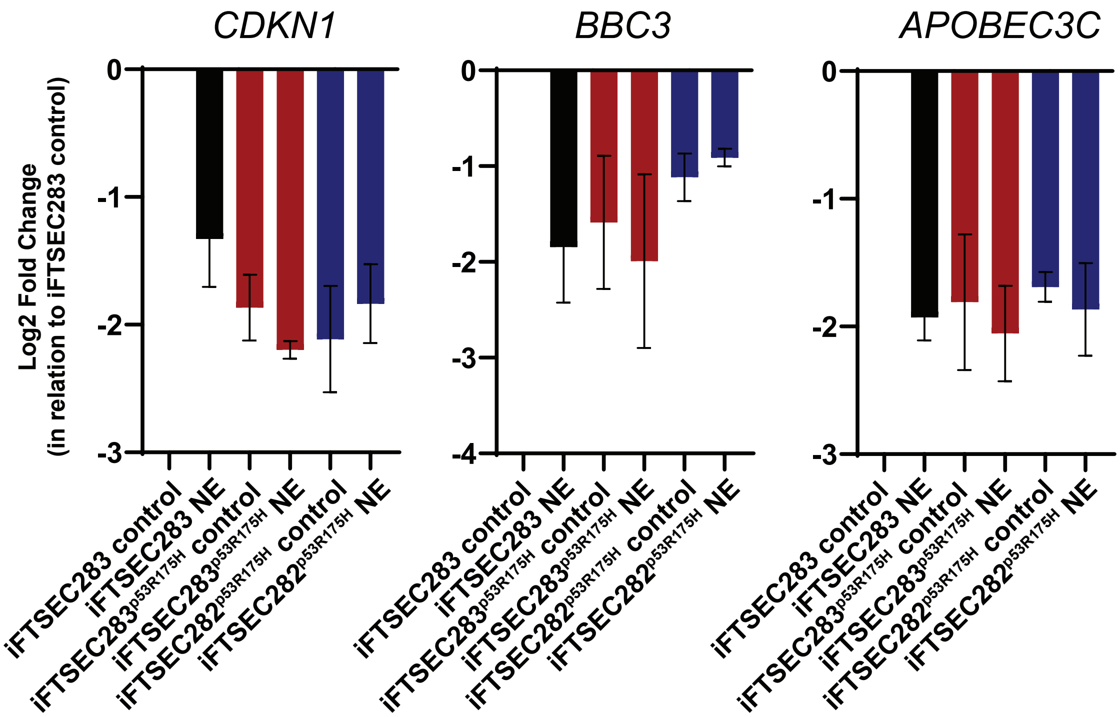

Supplementary Figure 4. Comparative expression of *TP53* target genes *CDKN1*, *BBC3*, and *APOBEC3*. Gene expression is plotted in reference to the iFTSEC283 control (no NE).
